# Long-term efficacy and safety of nalfurafine hydrochloride on pruritus in chronic liver disease patients: Patient-reported outcome based analyses

**DOI:** 10.1371/journal.pone.0178991

**Published:** 2017-06-12

**Authors:** Kenya Kamimura, Takeshi Yokoo, Hiroteru Kamimura, Akira Sakamaki, Satoshi Abe, Atsunori Tsuchiya, Masaaki Takamura, Hirokazu Kawai, Satoshi Yamagiwa, Shuji Terai

**Affiliations:** Division of Gastroenterology and Hepatology, Graduate School of Medical and Dental Sciences, Niigata University, Asahimachido-ri, Chuo-ku, Niigata, Niigata, Japan; Chiba University, Graduate School of Medicine, JAPAN

## Abstract

**Background and aim:**

Among various symptoms accompanied with chronic liver disease, pruritus affects the quality of life of patients, causing physical and mental stress, and worsens hepatic function. Recently, *κ*-opioid receptor agonist, nalfurafine hydrochloride was approved to treat central pruritus in patients with liver disease in Japan. This study aimed to assess the long-term efficacy and safety of nalfurafine hydrochloride on pruritus in chronic liver disease patients.

**Methods:**

A patient-reported outcome using questionnaire-based methods was used for 41 liver disease patients with or without pruritus symptoms. Nalfurafine hydrochloride (2.5 μg/day) was orally administered to 18 patients suffering from pruritus symptoms and whose current treatment was not effective. The same questionnaires and visual analogue scales (VAS) were repeatedly followed up for the patients for the entire follow-up period, and biochemical analyses were performed to evaluate the safety of the treatment.

**Results:**

Pruritus completely disappeared in seven of 18 cases, and VAS scores showed a decreasing trend over time from the start of nalfurafine hydrochloride administration in all patients who received the medication. Among 11 patients who were followed up for more than 12 weeks, nine patients showed continuous improvement of symptoms, and this progress was still apparent at ≥20 weeks after starting administration (p < 0.0001). The medication was discontinued in four patients because of progression of primary disease, high cost, oral dryness, and anemia. No significant toxicity was observed on the serum biochemical analyses.

**Conclusions:**

Nalfurafine hydrochloride contributed to long-term suppression of pruritus without significant safety problems.

## Introduction

Many cases of chronic liver disease are accompanied by pruritus caused by cholestasis, which cause systemic itching without any skin lesions. The symptoms cause both physical and mental stress and affect the quality life (QOL) of the patients. The mechanism of pruritus includes upregulation of the *μ*-opioid receptor system and suppression of the *κ*-opioid receptor system. Therefore, anti-histamines and sedatives show insufficient effects. Pruritus has also been seen and extensively studied in hemodialysis patients [1] and recently, therapeutic effect of *κ*-opioid receptor agonist, nalfurafine hydrochloride, on the itching by modulating the central nervous signals, have been reported in the hemodialysis patients [2, 3].

Because the same mechanisms are involved in liver disease-related itching which affect their QOL [4–7], the administration of nalfurafine hydrochloride in chronic liver disease patients has recently been approved in Japan based on a randomized, double-blind trial assessing the efficacy of nalfurafine hydrochloride in patients with liver disease [8]. The efficacy of nalfurafine hydrochloride was evidenced by the administration of 2.5 or 5 μg daily for 12 weeks, assessing the changes of pruritus by a visual analogue scale (VAS) and pruritus score. In terms of long-term efficacy of nalfurafine hydrochloride, Kumagai H et al. reported that time-dependent improvement of symptoms assessed by VAS was seen for 1 year of observation, and long-term suppression of pruritus was evidenced in their clinical study for patients on hemodialysis [2]; however, no studies of nalfurafine hydrochloride in patients with liver disease have been reported to date.

Therefore, we conducted this study to assess the long-term efficacy and safety of nalfurafine hydrochloride on pruritus in chronic liver disease patients.

## Methods

### Study design

This study was designed as a non-interventional proportional study with patient-reported, questionnaire-based outcomes. All the protocols were approved by the ethics committee and institutional review board of Niigata University School of Medicine Institutional Review. Written informed consent was obtained from all patients, and the study was conducted in accordance with the ethical guidance of the 1975 Declaration of Helsinki. The study included non-pruritus and pruritus patients and the participant recruitment started in Nov, 2015. The patients with chronic liver diseases treated in our hospital regularly and who can continuously answer the questionnaires (S1 and S2 Texts) have been included, and the patients who are unable to answer by their own or unable to visit us regularly have been excluded. The same questionnaires were repeatedly used on the day of appointments during the entire follow-up period to monitor symptoms in patients treated with nalfurafine hydrochloride (2.5 μg/day oral administration).

### Evaluation of pruritus

We evaluated pruritus using VAS and by patient answers reported on the questionnaires. The VAS value was a 100-mm horizontal line, in which its left end (0 mm) represented no pruritus and the right end (100 mm) represented maximum pruritus. All patients marked the point on the scale corresponding to their highest severity of pruritus during the last 12 h in the absence of observation by physicians or other staff [9]. The values were recorded on all appointment days during the follow-up period, with the interval ranging from 2 to 8 weeks, and the difference from the value recorded at the previous appointment was calculated to assess any change in symptoms. The questionnaires included a survey to evaluate the degree of itchiness, timing of itchiness, whether oral or topical medications were being used, and the degree of effects observed by the subjects who were administered nalfurafine hydrochloride [8]. The interviews regarding the degree of itchiness included choices of “Sometimes my hand moves to lightly scratch myself,” “I feel quite itchy and scratch even in public,” and “I feel so itchy that I can’t stand it.” The questionnaire data were compared for all time points, and the trend was analyzed in a time-dependent manner. Addiction liability was monitored by an in-charge physician interviewing patients at every appointment.

### Safety assessment

Safety was evaluated by assessing adverse events (AEs) using subjective and objective symptoms, vital signs (body temperature, blood pressure, and heart rate), and hematological and serum biochemical exams monitored at each appointment for the entire follow-up period after the initiation of nalfurafine hydrochloride. The NCI-CTCAE (National Cancer Institute Common Terminology Criteria for Adverse Events) version 4.0 was used to assess the AE of the therapies.

### Statistical analysis

The intergroup comparison of non-pruritus and pruritus patients was performed using the Mann–Whitney–Wilcoxon test. The change in VAS values from the first analyses were plotted for each exact time point after the start of treatment for all patients, and the trend was analyzed using Pearson’s correlation test. The time-dependent changes of serum biochemical assays were analyzed using Pearson’s correlation test. P-values of less than 0.05 were considered significant.

## Results

### Presence/Absence of pruritus

A total of 41 patients who meet the inclusion criteria completed the questionnaires regarding the existence of pruritus, and we found that 18 chronic liver disease patients appeared to be experiencing pruritus. While the intergroup comparison indicated no statistically significant differences for age, sex, presence/absence of liver cirrhosis, Child–Pugh score, and other biochemical markers, underlying primary biliary cholangitis (PBC) was significantly more common in the pruritus group than in the non-pruritus group (p = 0.0013). A tendency for lower hemoglobin (Hb) values was also observed in the pruritus group (p = 0.0082) **(Table 1)**.

**Table 1:** Patient characteristics for the survey.

Itching	-	+	MWW Test	Total
	n = 23	n = 18	*P*-value	n = 41
Characteristics				
**Age (years)**			0.28	
**Median**	67.0	69.0		68
**Range**	41–80	45–82		41–82
**Gender**			0.11	
**Female**	12	14		26
**Male**	11	4		15
**Etiology**			0.0013	
**HBV infection**	4	0		4
**HCV infection**	3	2		5
**Nonalcoholic steatohepatitis**	4	0		4
**Alcohol**	2	2		4
**Autoimmune hepatitis**	0	1		1
**Primary biliary cirrhosis**	6	11		17
**Primary sclerosing cholangitis**	1	0		1
**Idiopathic portal hypertension**	1	0		1
**Vanishing bile duct syndrome**	0	2		2
**Drug induced liver injury**	2	0		2
**Cirrhosis**			0.27	
**Yes/No**	17/6	17/1		34/5
**Child-Pugh Grade**			0.55	
**A/B/C**	16/1/0	15/1/1		31/2//1
**AST (IU/l)**			1.00	
**Median**	29.0	35.0		34.0
**Range**	19–153	22–95		19–153
**ALT (IU/l)**			0.29	
**Median**	24.0	24.0		24.0
**Range**	15–132	11–77		11–132
**T-Bil (mg/dl)**			0.24	
**Median**	0.7	0.9		0.8
**Range**	0.3–1.7	0.3–32.9		0.3–32.9
**D-Bil (mg/dl)**			0.16	
**Median**	0.1	0.1		0.1
**Range**	0.1–0.6	0.1–24.7		0.1–24.7
**Alb (g/dl)**			0.41	
**Median**	4.1	3.85		4.05
**Range**	2.5–4.5	2.1–4.5		2.1–4.5
**ChE (IU/l)**			0.59	
**Median**	268.0	286.0		272.0
**Range**	128–401	51–519		51–519
**PT-INR**			0.81	
**Median**	1.05	1.04		1.05
**Range**	0.97–1.33	0.92–1.29		0.92–1.33
**BUN (mg/dl)**			0.29	
**Median**	15.0	17.0		16.0
**Range**	11–30	6–35		6–35
**Crt (mg/dl)**			0.74	
**Median**	0.76	0.70		0.72
**Range**	0.36–1.61	0.38–1.84		0.36–1.84
**Hb (g/dl)**			0.0082	
**Median**	13.3	11.6		12.8
**Range**	9.7–16.2	8.3–15.7		8.3–16.2

HBV, hepatitis B virus; HCV, hepatitis C virus; AST, aspartate aminotransferase; ALT, alanine aminotransferase; T-Bil, total bilirubin; D-Bil, direct bilirubin; Alb, albumin; ChE, choline esterase; PT-INR, international normalized ratio of prothrombin time; BUN, blood urea nitrogen; Crt, creatinine; Hb, hemoglobin

### Detailed pruritus data

**Fig 1:**
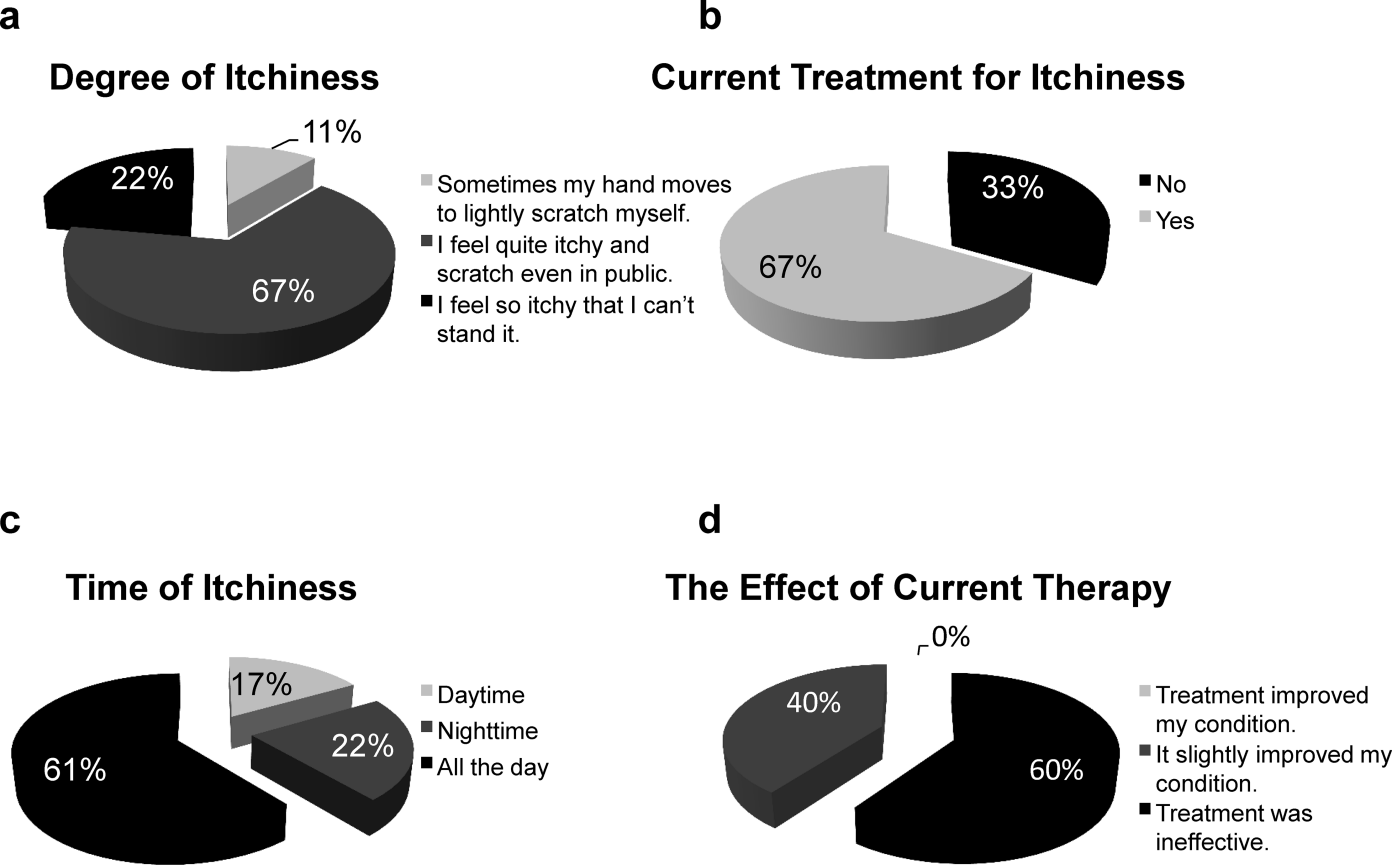
Pruritus in chronic liver disease patients (n = 18). **(a)** The degree of itchiness. **(b)** Treatment for the itchiness. **(c)** Time of itchiness. **(d)** The therapeutic effect of current therapy.

We used a survey to evaluate the degree of itchiness, timing of itchiness, whether oral or topical medications were being used, and the degree of effects of treatment in the 18 subjects who suffered from pruritus **(Fig 1)**. The degree of itchiness was as follows: “Sometimes my hand moves to lightly scratch myself” in 11%, “I feel quite itchy and scratch even in public” in 67%, and “I feel so itchy that I can’t stand it” in 22% of patients **(Fig 1A)**. We found that 67% of subjects were using oral or topical medication for itchiness, whereas 33% did not receive any medical interventions **(Fig 1B)**. The timing of itchiness was during the day in 17%, during the night in 22%, and both during the day and night in 61% of patients **(Fig 1C)**. Of the subjects using oral or topical medication, 0% answered “Treatment improved my condition,” 40% answered “It slightly improved my condition,” and 60% answered “Treatment was ineffective” **(Fig 1D)**.

### Effects of nalfurafine hydrochloride

**Fig 2:**
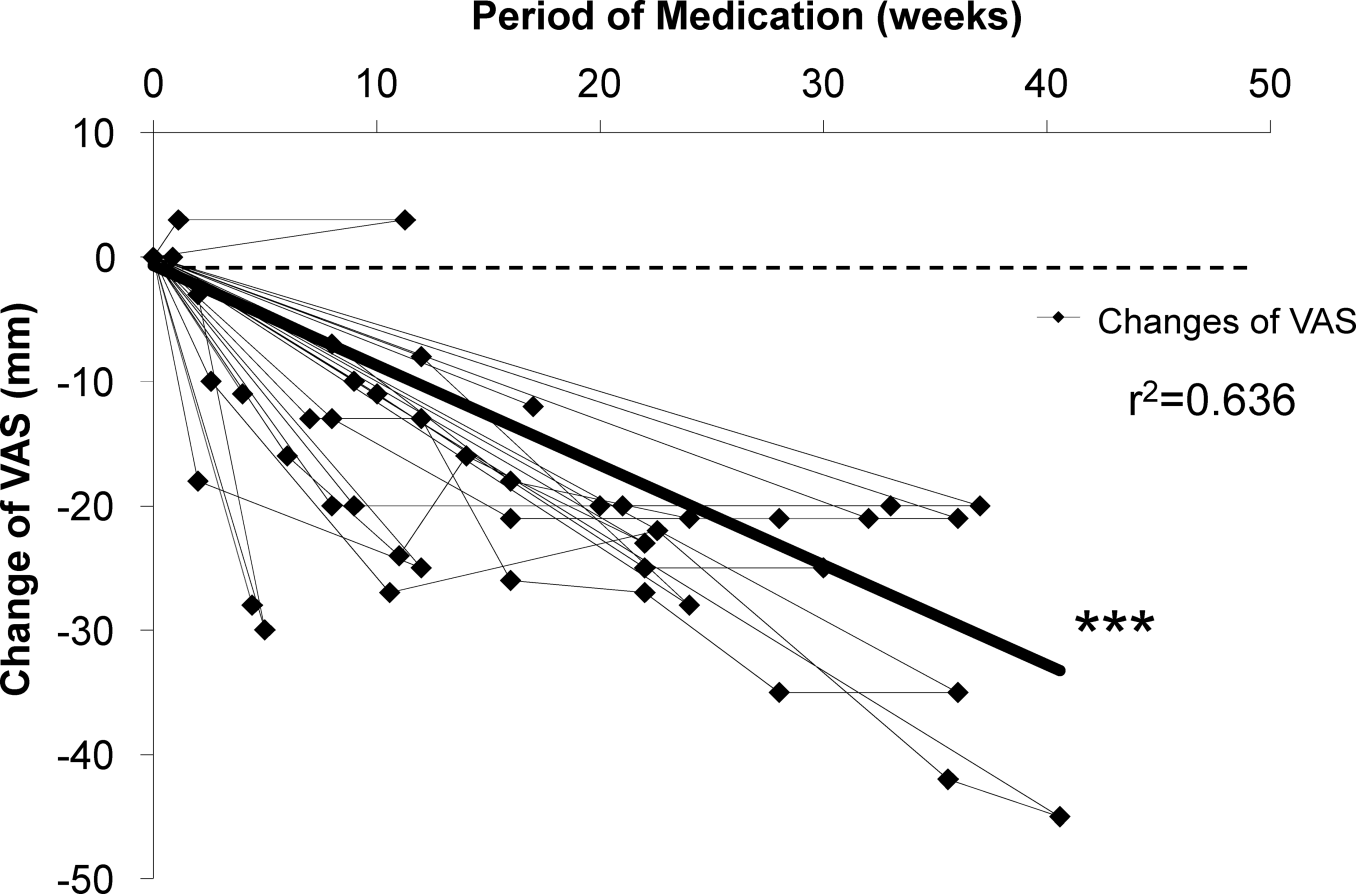
Time-dependent changes of symptoms measured by the visual analogue scale (VAS). The black lines with marks indicate all data from patients. The bold black line shows the trend line, and correlation analysis between change of VAS and period of medication was performed. *** for *p* < 0.001; r, correlation coefficient

We administered nalfurafine hydrochloride to 18 patients with pruritus. The medication was discontinued due to the progression of primary disease 1 week after the start of treatment (n = 1), high cost of treatment after 4 weeks of treatment (n = 1), and AEs, including oral dryness and anemia at 9 weeks and 17 weeks, respectively (n = 2) **(Table 2)**. The effects of the treatment were evaluated by patient-reported outcomes using VAS scores. Pruritus completely disappeared in seven cases (mean: 16 weeks, 4–41 weeks), and there was a decreasing trend over time from the start of administration in VAS scores of all patients who received the medication. Among 11 patients who were followed up for more than 12 weeks, two patients experienced complete disappearance of pruritus and the remaining nine patients showed further improvement of the symptoms based on the VAS scores. This improvement was still apparent at ≥20 weeks (p < 0.0001) **(Fig 2)**.

**Table 2:** Summary of patients treated with nalfurafine hydrochloride.

**Nalfurafine Hydrochloride (2.5 μg)**			
**Number of Patients**	18		
1	**Period of Medication (week)**		
**Median**	20.0		
**Range**	1–41		
Case (AE)	Symptoms/Reason	Time from Initiation (week)	Course
1	oral dryness	9	suspended
2	anemia	10	suspended
3	progression of disease	1	suspended
4	economical reason	4	suspended
5	urinary urgency	10	continued
6	insomnia	5	continued

AE, adverse event

**Fig 3:**
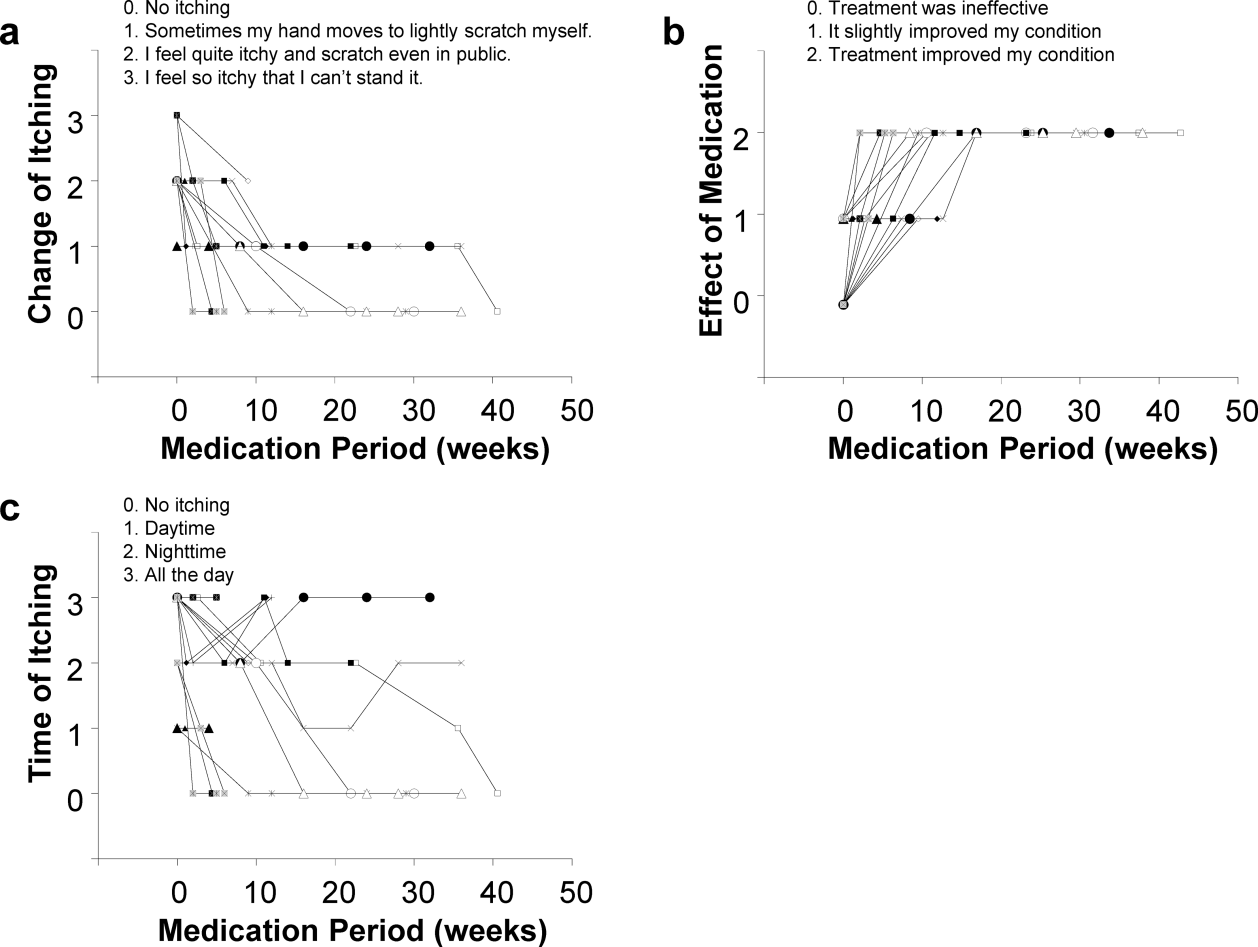
Results of long-term follow-up of the survey. **(a)** Change of itchiness. **(b)** Effect of medication. **(c)** Timing of itching.

The follow-up survey was performed on all 18 patients, and among 14 patients, excluding four patients in whom medication was discontinued because of the abovementioned reasons, seven showed complete disappearance of pruritus with no recurrence in the entire period of the study. The remaining seven patients showed significant improvement, and the degree of pruritus remaining was described as “Sometimes my hand moves to lightly scratch myself” **(Fig 3A)**. In addition, all seven patients subsequently reported the effects of the medication as “Treatment improved my condition” at ≥15 weeks after starting treatment **(Fig 3B)**. No trend for the timing of appearance of symptoms was observed in these seven patients **(Fig 3C)**. No patients showed recurrence of the symptoms. These results demonstrate the benefit of nalfurafine hydrochloride treatment on pruritus caused by chronic liver diseases.

### Safety of nalfurafine hydrochloride

AEs were seen in four patients and included oral dryness, anemia (CTCAE, grade 1), urinary urgency (CTCAE, grade 1), and insomnia (CTCAE, grade 1). The administration of nalfurafine hydrochloride was discontinued in two patients experiencing oral dryness and anemia, and their symptoms improved **(Table 2)**. The administration of nalfurafine hydrochloride was continued in two patients experiencing urinary urgency and insomnia because of the potential benefit of improving pruritus **(Table 2)**. And careful follow-up of biochemical findings for all cases, including the level of aspartate aminotransferase (AST), alanine aminotransferase (ALT), total bilirubin (T-Bil), direct bilirubin (D-Bil), albumin (Alb), choline esterase (ChE), and international normalized ratio of prothrombin time (PT-INR), were performed to monitor their hepatic function **(S1 Fig)**. Blood urea nitrogen (BUN), creatinine (Crt), and Hb were also monitored for potential AEs of nalfurafine hydrochloride treatment **(S2 Fig)**. The results of blood biochemical exams did not indicate any decrease in hepatic reserve function or any other AEs **(S1 and S2 Figs)**.

## Discussion

Pruritus is a symptom that can cause disruption of daily activities, sleep, and worsen QOL in patients. It is classified into two main types: peripheral and central. Peripheral pruritus is caused by the activation of mast cells releasing histamine through C fibers, and central pruritus is caused by the activation of *μ*-opioid receptors by various agonists, including β-endorphin, encephalin, and more. Scratching further activates C fibers and worsens the symptom for both types of pruritus. Central pruritus can be observed in patients on hemodialysis and in those with chronic liver diseases [7, 10, 11]; however, no effective treatments had been developed. It has been reported that chronic liver diseases are accompanied with pruritus, and it impairs activity and sleep, and causes malaise and cramping limbs, thereby affecting QOL [4–7, 10, 11]. It was recently reported that lysophosphatidic acid (LPA) and autotaxin (ATX), which is involved in the production of LPA, are involved in the development of central pruritus in cases of PBC [12]. More recently, the presence of ATX stimulating factors has also been reported [7]. Our study showed a higher rate of itching in PBC patients, likely due to this mechanism. The reasons for a relatively lower level of Hb in patients with pruritus remain unclear.

Because recent advances in pharmaceutical development have contributed to the improvement of underlying liver diseases, e.g., antiviral medications for viral hepatitis, the management of various symptoms related to these liver diseases is important to maintain QOL and hepatic reserve. For this purpose, efforts have been made to develop medicine that can control central pruritus in patients with liver disease.

The central pruritus is unrelated to peripheral itching signals, anti-histamine agents are ineffective; therefore, recently, a *κ*-opioid receptor agonist, nalfurafine hydrochloride, was approved in Japan and Korea to treat the symptoms in patients on hemodialysis based on its demonstrated efficacy [2, 3]. More recently, it was approved in Japan for central pruritus in chronic liver disease patients, and results of the clinical trial were reported [8]. The trial included 103, 105, and 109 patients who were treated with placebo, 2.5 μg, or 5 μg of nalfurafine hydrochloride, respectively, for 12 weeks, and the improvement of symptoms and AEs were carefully monitored. The study showed clinical therapeutic efficacy with an improvement of VAS, and no AEs were reported [8] for the entire study period. Based on those results, we further assessed the long-term efficacy and safety of nalfurafine hydrochloride by assessing VAS, questionnaire-based epidemiology, and biochemical findings to strengthen the evidence of the benefit of nalfurafine hydrochloride treatment of pruritus with chronic liver disease. We showed that the administration of nalfurafine hydrochloride improved pruritus symptoms after starting the therapy and over a long term. Among 11 patients receiving nalfurafine hydrochloride for more than 12 weeks, two patients experienced complete disappearance of pruritus and the remaining nine patients showed further improvement of the symptoms. One patient showed continuous improvement by 41 weeks and no patients had recurrence of the symptoms **(Fig 2)**.

And importantly, careful follow-up by serum biochemical analyses showed no significant AEs or worsening of the hepatic reserve functions. In addition, only two patients discontinued treatment because of symptoms of oral dryness and anemia **(Table 2)**. These results indicate the safety and efficacy of nalfurafine hydrochloride for the treatment of central pruritus in chronic liver disease patients.

**Fig 4:**
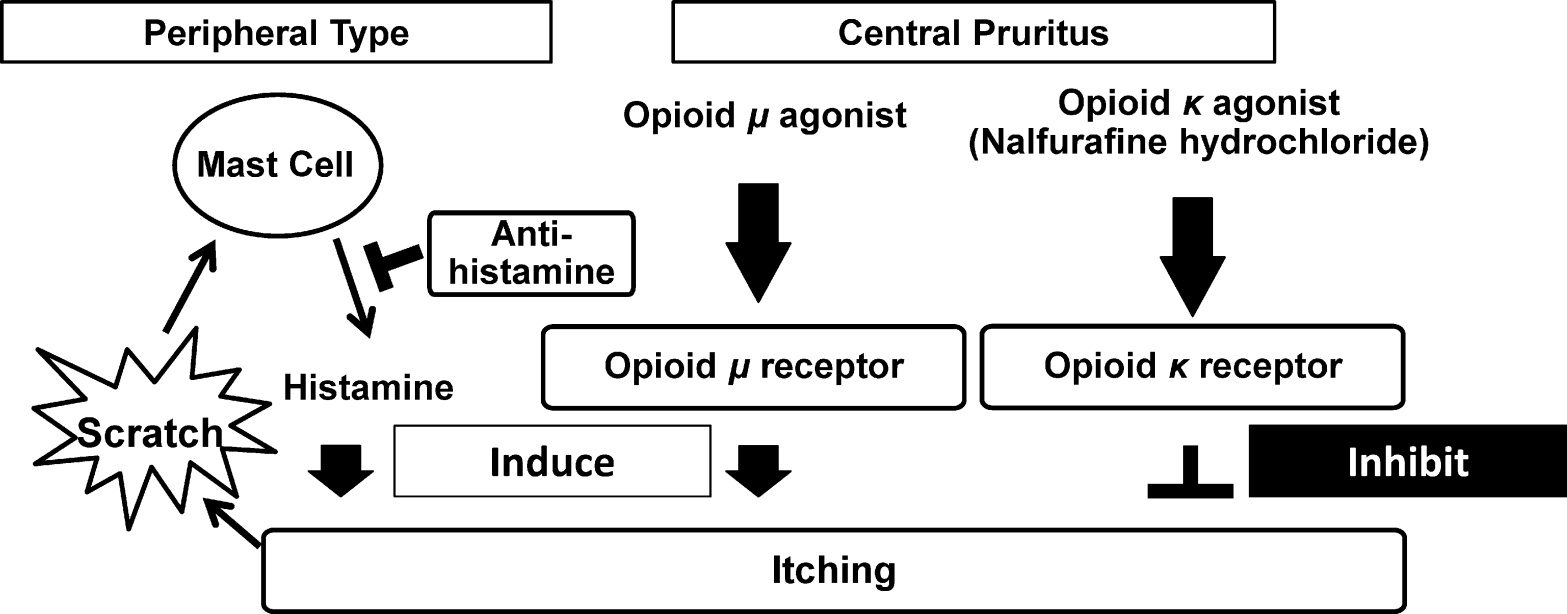
Mechanism of central pruritus in chronic liver disease.

The limitation of this study is that only a small number of patients were treated in this study. It is obvious that further studies are necessary to examine the effects of nalfurafine hydrochloride in a larger population and in other countries as it is currently approved only in Japan. In addition, because peripheral pruritus is activated by scratching, inducing neuropeptides from C fibers, thereby activating mast cells and increasing levels of histamine [13] **(Fig 4)**, combination therapy of nalfurafine hydrochloride with anti-histamines should be tested to evaluate the effect of the combination treatment on the itching. However, this is the first report showing the long-term efficacy and safety of nalfurafine hydrochloride for liver disease patients without any severe adverse events.

In summary, this prospective study showed long-term efficacy and safety of nalfurafine hydrochloride for the management of central pruritus. The maintenance of QOL and liver function could improve the long-term prognosis for chronic liver disease patients, although, so far, no evidence of improvement of hepatic function has been observed during this follow-up period.

## Supporting information

S1 Fig Time-dependent changes in serum biochemical data related to hepatic function. Data represent the changes from initial value. AST, aspartate aminotransferase; ALT, alanine aminotransferase; T-Bil, total bilirubin; D-Bil, direct bilirubin; Alb, albumin; ChE, choline esterase; PT-INR, international normalized ratio of prothrombin time; N.S., not significant; r, correlation coefficient. For PT-INR, 7 cases were excluded due to warfarinization.
(TIFF)

S2 Fig Time-dependent changes in serum biochemical data. Data represent the changes from initial value. BUN, blood urea nitrogen; Crt, creatinine; Hb, hemoglobin; N.S., not significant; r, correlation coefficient.
(TIFF)

S1 Text A copy of the questionnaire in English. (DOCX)

S2 Text A copy of the questionnaire in original language. (DOCX)

## References

[1] Kumagai H Saruta T, in Yosipovitch G Greaves MW.: Itch: Basic Mechanisms and Therapy. New York, Marcel Dekker, 2004, pp279-286.

[2] Kumagai H, Ebata T, Takamori K, Muramatsu T, Nakamoto H, Suzuki H. Effect of a novel kappa-receptor agonist, nalfurafine hydrochloride, on severe itch in 337 haemodialysis patients: a Phase III, randomized, double-blind, placebo-controlled study. Nephrol Dial Transplant. 2010; 25: 1251–1257. doi: 10.1093/ndt/gfp588 19926718 10.1093/ndt/gfp588

[3] Kumagai H, Ebata T, Takamori K, Miyasato K, Muramatsu T, Nakamoto H et al Efficacy and safety of a novel k-agonist for managing intractable pruritus in dialysis patients. Am J Nephrol. 2012; 36: 175–183. doi: 10.1159/000341268 22868684 10.1159/000341268

[4] Jones EA, Bergasa NV. The pruritus of cholestasis: from bile acids to opiate agonists. Hepatology. 1990; 11: 884–887. 2161397 10.1002/hep.1840110526

[5] Jones EA, Bergasa NV. The pruritus of cholestasis. Hepatology. 1999; 29: 1003–1006 doi: 10.1002/hep.510290450 10094938 10.1002/hep.510290450

[6] Bergasa NV. Pruritus and fatigue in primary biliary cirrhosis. Clin Liver Dis. 2003; 7: 879–900. 14594135 10.1016/s1089-3261(03)00105-3

[7] Beuers U, Kremer AE, Bolier R, Elferink RP. Pruritus in cholestasis: facts and fiction. Hepatology. 2014; 60: 399–407. doi: 10.1002/hep.26909 24807046 10.1002/hep.26909

[8] Kumada H, Miyakawa H, Muramatsu T, Ando N, Oh T, Takamori K et al Efficacy of nalfurafine hydrochloride in patients with chronic liver disease with refractory pruritus: A randomized, double-blind trial. Hepatol Res. 2016 10 18 2016; doi: 10.1111/hepr.12830 27753159 10.1111/hepr.12830

[9] Wahlgren CF, Ekblom A, Hagermark O. Some aspects of the experimental induction and measurement of itch. Acta Derm Venereol. 1989; 69: 185–189. 2566219

[10] Gillespie DA, Vickers CR. Pruritus and cholestasis: therapeutic options. J Gastroenterol Hepatol. 1993; 8: 168–173. 8471755 10.1111/j.1440-1746.1993.tb01510.x

[11] Suzuki K, Tamano M, Katayama Y, Kuniyoshi T, Kagawa K, Takada H et al Study of pruritus in chronic hepatitis C patients. World J Gastroenterol. 2014; 20: 17877–17882. doi: 10.3748/wjg.v20.i47.17877 25548485 10.3748/wjg.v20.i47.17877 PMC4273137

[12] Kremer AE, Martens JJ, Kulik W, Rueff F, Kuiper EM, van Buuren HR et al Lysophosphatidic acid is a potential mediator of cholestatic pruritus. Gastroenterology. 2010; 139: 1008–1018. doi: 10.1053/j.gastro.2010.05.009 20546739 10.1053/j.gastro.2010.05.009

[13] Umeuchi H, Togashi Y, Honda T, Nakao K, Okano K, Tanaka T et al Involvement of central mu-opioid system in the scratching behavior in mice, and the suppression of it by the activation of kappa-opioid system. Eur J Pharmacol. 2003; 477: 29–35. 14512095 10.1016/j.ejphar.2003.08.007

