# Supplementary material for: Long-term efficacy and safety of nalfurafine hydrochloride on pruritus in chronic liver disease patients: Patient-reported outcome based analyses
Source: PLoS One. 2017 Jun 12;12(6):e0178991. doi: 10.1371/journal.pone.0178991 (PMC5467861; doi:10.1371/journal.pone.0178991)
Supplement: S1 Text — (DOCX) [file pone.0178991.s003.docx]

**S3 Text**

**A copy of the survey questions or questionnaire**

1. **Do you feel itchiness?**

**Yes/No**

1. **Degree of itchiness**
2. **Sometimes my hand moves to lightly scratch myself.**
3. **I feel quite itchy and scratch even in public.**
4. **I feel so itchy that I can’t stand it.**
5. **Current Treatment for Itchiness**

**Yes/No**

1. **Time of itchiness**
2. **Daytime**
3. **Nighttime**
4. **All the day**
5. **The effect of current therapy**
6. **Treatment improved my condition.**
7. **It slightly improved my condition.**
8. **Treatment was ineffective.**
9. **VAS score**

**Plot the point on the scale below corresponding to your highest severity of pruritus during the last 12 h.**

**(left side end, no itchiness; right side end, maximum itchiness you have felt)**
