# Supplementary material for: Long-term efficacy and safety of nalfurafine hydrochloride on pruritus in chronic liver disease patients: Patient-reported outcome based analyses
Source: PLoS One. 2017 Jun 12;12(6):e0178991. doi: 10.1371/journal.pone.0178991 (PMC5467861; doi:10.1371/journal.pone.0178991)
Supplement: S2 Text — (DOCX) [file pone.0178991.s004.docx]

**S4 Text**

**A copy of the survey questions or questionnaire (in original language)**

1. **現在かゆみを感じますか。**

**はい/いいえ**

1. **かゆみの程度はどのくらいですか。**
2. **ときに手がゆき、軽くかく。**
3. **かなりかゆく、人前でもかく。**
4. **いてもたってもいられない。**
5. **現在、かゆみに対するお薬を使用していますか。**

**はい/いいえ**

1. **いつ、かゆいですか。**
2. **昼**
3. **夜**
4. **一日中**
5. **現在、かゆみに対するお薬を使用している方に：その効果はいかがですか。**
6. **よくなる。**
7. **すこしよくなる。**
8. **よくならない。**
9. **VAS score**

**この半日で、もっともかゆかったときの程度を下の線の上に印をつけてください。**
